# Evaluating the Usefulness of a Large Language Model as a Wholesome Tool for De Novo Polymerase Chain Reaction (PCR) Primer Design

**DOI:** 10.7759/cureus.47711

**Published:** 2023-10-26

**Authors:** Soham Jorapur, Amisha Srivastava, Suyamindra Kulkarni

**Affiliations:** 1 Department of Biological Sciences, Indian Institute of Science Education and Research, Bhopal, Bhopal, IND; 2 Department of Electrical & Computer Engineering, University of Texas at Dallas, Richardson, USA; 3 Department of Higher Education, Government of Karnataka, Karnataka Institute for DNA Research (KIDNAR), Dharwad, IND

**Keywords:** monkeypox virus, large language models (llms), primer, chat gpt, molecular biology, pcr, diagnostics

## Abstract

This study aimed to assess the ability of language learning models (LLMs), specifically GPT-3.5 (Chat Generative Pre-trained Transformer 3.5) and GPT-4 (Chat Generative Pre-trained Transformer 3.5), in designing primers for diagnostic polymerase chain reaction (PCR) of the monkeypox virus (MPXV). Five primer pairs were generated by each LLM, and their thermodynamic properties and specificity were analysed post-hoc using commonly used software. The LLMs demonstrated ability in sequence generation and predicting melting temperatures (Tm), but their accuracy in predicting GC content was suboptimal, necessitating further investigation. Results indicated that, of the total primer pairs, only three designed by GPT-4 and two by GPT-3.5 could theoretically form a PCR product, but only one pair demonstrated suitable parameters for experimental validation. This preliminary exploration suggests that while LLMs have a potential in aiding primer design, their accuracy needs improvement to match current deterministic, rule-based tools used in the field. Consequently, manual intervention remains a crucial step in PCR primer design.

## Introduction

Polymerase chain reaction (PCR) is a transformative molecular biology technique that has revolutionised the field of genetic analysis and research. Developed in the mid-1980s by Kary B. Mullis, PCR enables the targeted and exponential amplification of specific DNA sequences, making it an essential tool in various scientific disciplines. Its tremendous impact on the scientific community was recognised with the prestigious 1993 Nobel Prize in Chemistry [1]. The fundamental principle of PCR lies in its ability to repeatedly copy and amplify a specific DNA segment, even from a minute quantity of starting material. The PCR process involves a carefully orchestrated series of temperature cycles, each consisting of denaturation, annealing and extension steps. During denaturation, the DNA strands are separated by heating, and in the subsequent annealing step, especially designed short DNA sequences called primers bind to the complementary regions flanking the target DNA sequence. These primers act as starting points for DNA synthesis [2].

The versatility and applicability of PCR are reflected in its wide range of applications. In the field of molecular diagnostics, PCR has become an indispensable tool for the detection of various genetic diseases and pathogens. Its sensitivity and specificity make it particularly suited for accurate and early diagnosis. PCR is also heavily employed in genetic engineering and cloning, allowing scientists to create multiple copies of a specific DNA fragment, essential for gene manipulation and genetic studies [3].

However, one of the critical challenges in the PCR process is the design of primers. The success of PCR heavily relies on the proper design of these short DNA sequences. Primers need to be highly specific to the target DNA sequence to avoid non-specific amplification, which can lead to misleading results or contamination. In addition, the melting temperature (Tm) of the primers must be carefully considered to ensure optimal annealing during the temperature cycles [4]. To address the complexities of primer design, various software tools have been developed to aid researchers in selecting appropriate primers for specific PCR applications. These tools offer algorithms that consider factors, such as primer length, GC content, Tm, secondary structures and primer-dimer formation to optimise primer specificity and efficiency.

The potential of advanced language models, such as ChatGPT-3.5 (Chat Generative Pre-trained Transformer 3.5) and GPT-4 (Generative Pre-trained Transformer 4) developed by OpenAI [5], to streamline and enhance primer design in PCR represents a promising frontier in molecular biology research. These language models, based on large language models (LLMs), have showcased remarkable natural language processing capabilities, capable of generating human-like text and answering questions based on provided prompts and context [6]. This study aims to explore the integration of ChatGPT-3.5 and GPT-4 in the process of automated primer design for various PCR techniques. By leveraging the knowledge and context provided by these advanced language models, we aim to improve the efficiency and accuracy of primer design, thereby advancing the field of PCR research and development.

To achieve this goal, we have used a comprehensive dataset comprising experimentally validated primer and target DNA sequences sourced from viral disease diagnostic data. These sequences encompass a diverse range of PCR applications, providing a robust foundation for evaluating the performance of the language models in primer design. Our approach involves subjecting ChatGPT-3.5 and GPT-4 to this dataset and conducting a pre-training evaluation to validate their understanding of the provided molecular biology data. Practical questions designed for various PCR applications are posed to each model, allowing us to assess their ability to generate appropriate primer sequences for specific DNA targets. The comparison between the primer designs generated by ChatGPT-3.5, GPT-4 and current manual methods will provide valuable insights into the effectiveness and potential of integrating advanced language models in PCR research. Furthermore, this exploration of artificial intelligence's role in molecular biology raises important considerations about ethics, bias and control, highlighting the need for responsible and transparent use of such powerful tools.

As we delve into this intersection of advanced language models and molecular biology, we anticipate that our findings will not only optimise primer design but also pave the way for future advancements in PCR technology. Nevertheless, we recognise the ongoing efforts to address the challenges associated with artificial intelligence, and we are committed to ensuring the responsible and ethical use of these tools in scientific research.

## Materials and methods

This paper explores two prompt engineering models: few-shot prompting and chain-of-thought prompting for validating the use of ChatGPT as a primer designing tool.

Benchmarks

A curated dataset of experimental primer sequences was utilised for training purposes [7]. This dataset comprised primer details, including primer sequences, melting temperatures (Tm), GC content and other relevant parameters, along with their corresponding target sequences. The dataset was pruned to ensure diversity and representation of different primer characteristics. 

Few-shot prompting 

We adopt the widely used method of few-shot prompting [8]. In this approach, a language model is primed using in-context examples comprising pairs of input and output prior to generating predictions for a novel, unseen test instance. The in-context examples, known as 'exemplars', are structured in the form of a table. This tabular representation includes various known values and also consists of blank entries, which the model is tasked to complete or predict. During the prediction phase, the model generates answers directly, without the need for further human intervention or post-processing. This process emulates the human cognitive function of extrapolating information from known instances and applying this knowledge to novel situations. By utilising the few-shot prompting paradigm, our research leverages the model's inherent capacity to comprehend context, recognize patterns and generate accurate predictions based on its pre-existing training.

All models were posed with generating primers for DNA-dependent RNA polymerase of MPXV (monkeypox virus) (GenBank OR209312). To validate the effectiveness and accuracy of the generated primer sequences, in silico PCR analysis was performed using suitable software tools. This simulation-based approach assessed whether the generated primers successfully amplified the target regions as intended. The results of the in-silico PCR analysis provided insights into the feasibility and functionality of the primers generated by ChatGPT.

**Figure 1:**
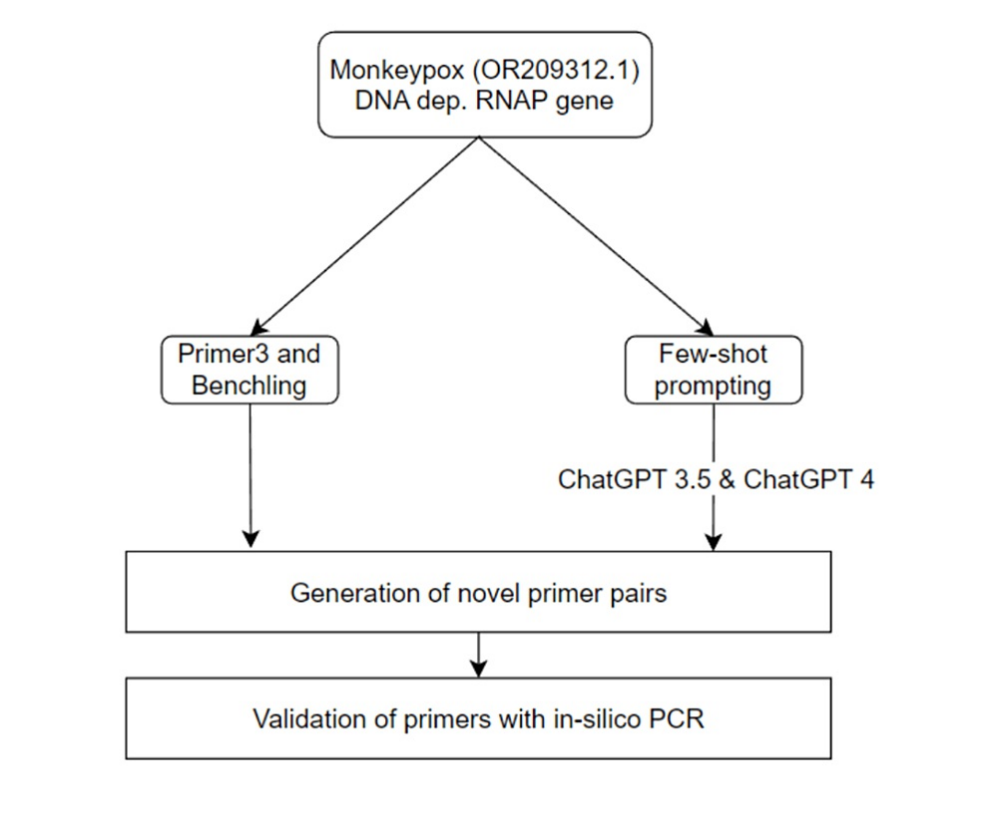
A DNA-dependent RNA polymerase (RNAP) of MPXV (monkeypox virus; GenBank OR209312.1) was selected as the target sequence for this study. We utilised a comprehensive dataset that included diverse primer details for training purposes. Using the few-shot prompting method, which leverages in-context examples allowing the model to extrapolate information, ChatGPT-3.5 and ChatGPT-4 were tasked with predicting novel primer sequences. In parallel, primers were also generated using contemporary standard software. To evaluate the effectiveness of the primers generated, an in-silico PCR analysis was carried out. Furthermore, the accuracy and reliability of these primers were ascertained confirming their specificity to the MPXV.

For primers that led to a PCR product prediction, further analysis of specificity and sensitivity was done. To ensure the accuracy and reliability of our primers, we validated them using various tools, such as Primer-BLAST (Primer- Basic Local Alignment Search Tool) and BLAST (Basic Local Alignment Search Tool). These tools enabled us to thoroughly investigate the amplification targets of the primers, ensuring that they were highly specific to the MPXV and sensitive enough to detect even small amounts of the virus in patient samples. A flowchart of an overview of the methods used is presented in Figure 1.

## Results

Five pairs of primers were generated using the three methods as described in the methodology. For sequences generated from LLM models, outputs were in the form of training data and did not include ΔG (Gibbs free energy change) and specificity calculations. These were calculated separately using the software Benchling (Benchling Inc., USA) [9]. It is interesting to note that while three out of the five primer pairs generated by ChatGPT-4 could theoretically form a PCR product, the number is two for ChatGPT-3.5

We present the results in a tabulated form in Table 1. A description of each column header is given: 1) *Primer pair no.*: the numeric identification used to distinguish between different primer pairs in a study; 2) *Generated by*: the software or method utilised to design and optimise the primer sequences; 3) *Sequence (5′ → 3′)*: the linear sequence of nucleotide bases in a primer, written from the 5' (five prime) end to the 3' (three prime) end; 4) *No. of mismatched bases*: the number of nucleotide bases that do not perfectly complement the template sequence; 5) *Size [nt]*: the length of the primer sequence, measured in nucleotides (nt); 6) *Purine content (GC %)*: the percentage of guanine (G) and cytosine (C) bases in the primer sequence, which can influence primer binding stability; 7) *Tm (°C)*: the melting temperature (Tm) of the primer, which is the temperature at which half of the primer-template duplexes have dissociated into single strands; 8) *ΔG homodimer (kcal)*: the Gibbs free energy change (ΔG) for the formation of homodimers, self-binding structures formed by two identical primers; 9) *ΔG monomer (kcal)*: the Gibbs free energy change (ΔG) for the formation of a monomer, or single-stranded structure of a primer; 10) *ΔG heterodimer (kcal)*: the Gibbs free energy change (ΔG) for the formation of heterodimers, structures formed by two different primers; 11) *Specificity for monkeypox (BLAST taxid: 10244) (%)*: the percentage indicating primer sequence specificity for MPXV, as determined by the BLAST algorithm; and 12) *Length of hypothetical Amplicon (bp)*: the size of the DNA fragment that would be produced in a PCR reaction using the primers, measured in base pairs (bp).

**Table 1:** Primer design results. Each row represents a distinct primer pair. The columns detail the primer's ID, creation method, nucleotide sequence, mismatches, length, GC content, melting temperature and potential self-binding energies (ΔG for homodimers, monomers and heterodimers). In addition, the table highlights each primer's specificity to the monkeypox virus and the expected size of the DNA fragment produced in the polymerase chain reaction (PCR). Values appear as i/j, where 'i' denotes the language model's prediction while 'j' the references values from the primer3 software.

Primer pair no.	Generated by	Sequence (5′ → 3′)	No. of mismatched bases	Size (nt)	Purine content (GC %)	Tm (°C)	ΔG homodimer (kcal)	ΔG monomer (kcal)	ΔG heterodimer (kcal)	Specificity for monkeypox (BLAST taxid: 10244) (%)	Length of hypothetical amplicon [bp]
1	Primer3	TCTCTGTGTCAGAACGCTCGTCA	0	23	52.17	59.7	-3.11	0	-4.14	100	1142
		TTGTGCTGCTCTTATCGTCTGA	0	22	45.45	55.9	-3.13	0		100	
2		CTCTGTGTCAGAACGCTCGTCA	0	22	54.55	58.7	-3.11	0	-4.14	100	1141
		TTGTGCTGCTCTTATCGTCTGA	0	22	45.45	55.9	-3.13	0		100	
3		TCTCTGTGTCAGAACGCTCGTCA	0	23	52.17	59.7	-3.11	0	-4.14	100	1141
		TGTGCTGCTCTTATCGTCTGAT	0	22	45.45	55.7	-3.13	0		100	
4		CTCTGTGTCAGAACGCTCGTCA	0	22	54.55	58.7	-3.11	0	-4.14	100	1140
		TGTGCTGCTCTTATCGTCTGAT	0	22	45.45	55.7	-3.13	0		100	
5		TCTCTGTGTCAGAACGCTCGTC	0	22	54.55	58.5	-3.11	0	-4.14	100	1142
		TTGTGCTGCTCTTATCGTCTGA	0	22	45.45	55.9	-3.13	0		100	
1	ChatGPT-4	TCTCTGTGTCAGAACGCTCG	0	20	50 / 55	60 / 56.3	-3.11	0	-4.13	100	155
		CGGTTAATCAGAGCTACATTC	1	21	47.62 / 42.86	60.6/ 50.6	-3.25	0		100	
2		TGTTGACTCTCTTATCGTCTG	0	21	47.62 / 44.44	60.6 / 51.2	-1.83	0	-1.99	100	819
		CTAGAGCCGCTGATGAACCT	0 (11 nt long overhang)	20	50 / 55	60 / 16.3	-4.55	0		100	
3		CTCGTCAATATAGATCTTAG	5	20	45 / 35	58 / 35	-2.87	0	-2.42	100	59
		AACTCTCTCTAAAAAAAAATTCT	7	21	23.81 / 21.74	49.2 / 46.94	-1.45	0		100	
4		CGTTCTCGACACAGAGAGA	0	19	52.63 / 52.63	57.6 / 52.9	-3.64	0	-2.03	100	The reverse primer binds before the forward primer.
		GCACAGAGATTCTTCCAGATA	2	21	47.62 / 42.86	60.6 / 51.1	-2.12	0		100	
5		AAATTGGGAGGCTTAAAGTG	3	20	45 / 40	58 / 50.1	-5.19	0	-3.88	18	Complementarity on the reverse strands
		GTTTACCGTCCATGCCACAC	11	20	50 / 55	60 / 56	-3.63	0		0	
1	ChatGPT-3.5	TCTCTGTGTCAGAACGCTCG	0	20	50 / 55	56.5 / 56.	-3.11	0	-5.35	100	86
		CGTAGTTGCGTTAGTTCTCT	2	20	45 / 45	54.5 / 51.8	-1.57	0		95	
2		GATCTTAGAAATTTTTTAGA	5	20	45 / 20	54.5 / 40.1	-3.53	0	-2.12	1	Both primers are forward primers.
		TCTCTGTGTCAGAACGCTCG	0	20	50 / 55	56.5 / 56.	-3.11	0		100	
3		ATCATTCTTTTCCTCTTGAG	8	20	45 / 35	54.5 / 46.8	-1.7	0	-1.54	0	The reverse primer binds before the forward primer.
		CGTAGTTGCGTTAGTTCTCT	2	20	45 / 45	54.5 / 51.8	-1.57	0		95	
4		AAAGAATTCGAATCAAAGATA	5	20	45 / 23.81	54.5 / 45.1	-5.17	0	-3.11	0	The forward primer binds to the - strand.
		TCTCTGTGTCAGAACGCTCG	0	20	50 / 55	56.5 / 56.	-3.11	0		100	
5		TCTCTGTGTCAGAACGCTCG	0	20	50 / 55	56.5 / 56.	-3.11	0	-2.89	100	132
		AGAGGATGATGAATAAAATA	1	20	45 / 25	54.5 / 42.6	-2.27	0		0	

When two values are given in the form of *i/j*, *i* represents the LLM’s prediction while *j* is the value determined by the software primer3 [10-12].

## Discussion

Since all models were tasked with generating primers for the DNA-dependent RNA polymerase of MPXV (GenBank OR209312), the results primarily provide insights into this specific context. Nevertheless, the data garnered from this focused study still offer valuable preliminary insights into the capabilities and boundaries of LLMs in primer design for specific genomic targets.

Both models of ChatGPT could grasp that a primer is a short oligomer and made reasonable predictions of Tm. However, predictions of GC%, which could have been an easy task for an LLM of this order, was surprisingly not up to the mark. We suggest further studies with chain-of-thought prompting that might address this issue. The crux of this method lies in the assumption that the language model should not only generate accurate predictions but also rationalise these predictions logically, rooted in the context provided by the exemplars [8]. Each exemplar in chain-of-thought prompting serves a dual purpose. Firstly, it informs the model about the nature of the task, much like in the case of standard few-shot prompting. However, more importantly, it also acts as an explanatory guide, illuminating the reasoning process that leads to a particular output [13].

As for the primer pairs generated, on evaluation by in-silico PCR, we see that ChatGPT-4 performs marginally better than ChatGPT-3.5. However, only one primer pair is found to have parameters suitable for experimental validation. The ~6 °C Tm difference will prove to be a challenge for setting annealing and extension temperatures, and as is done in the case of using currently employed tools, manual tweaking of the sequences to achieve optimal/parameters will have to be done.

## Conclusions

This study has successfully tested two commonly used LLMs for designing primers for diagnostic PCR. The models were fed training data using few-shot prompting and were asked to generate five primers for the amplification-based detection of the MPXV (GenBank: OR209312.1). It was found that out of 10 AI-generated primer pairs, only one was found comparable enough to currently used rule-based, deterministic programs employed by researchers and professionals. We conclude that ChatGPT is not (yet) ready to directly help biologists and clinicians with PCR designing and that this process may still have to be done (at least partially) manually for a few years to come.
